# Astragalin Exerted Hypoglycemic Effect by Both Inhibiting α-Glucosidase and Modulating AMPK Signaling Pathway

**DOI:** 10.3390/nu17030406

**Published:** 2025-01-23

**Authors:** Qian Li, Zhangchang Yang, Huijie Lu, Fan Liu, Donglai Zhou, Yuxiao Zou

**Affiliations:** 1 Sericultural & Agri-Food Research Institute, Guangdong Academy of Agricultural Sciences, Key Laboratory of Functional Foods, Ministry of Agriculture and Rural Affairs, Guangdong Key Laboratory of Agricultural Products Processing, Guangzhou 510610, China; liq@gdaas.cn (Q.L.); yzx140484@163.com (Z.Y.); liufan@gdaas.cn (F.L.); zhoudonglai.2007@163.com (D.Z.); 2 Guangdong Laboratory for Lingnan Modern Agriculture, Maoming 525000, China; 3 College of Food Science, South China Agricultural University, Guangzhou 510642, China; 4 Institute of Animal Science, Guangdong Academy of Agricultural Sciences, Guangzhou 510640, China; luhuijie@mail2.sysu.edu.cn

**Keywords:** astragalin, α-glucosidase, inhibitory mechanism, molecular docking, AMPK pathway

## Abstract

Background: The hypoglycemic activity of mulberry leaf polyphenols has been widely studied, while its mechanism of action needs further elucidation. Methods: The inhibitory activity mechanism of astragalin on α-glucosidase was investigated with a combination of multispectroscopic techniques and molecular docking. The hypoglycemic pathway was further revealed with a high-glucose human hepatocellular carcinomas (HepG2) cell model. Results: The results indicated that astragalin inhibited α-glucosidase with IC_50_ of 154.5 µM, which was the highest in potency among the main polyphenols from mulberry leaves. Astragalin could bind to α-glucosidase with a single inhibition site and quench its endofluorescence with a static quenching mechanism. Astragalin changed the secondary structure of α-glucosidase, and the decreased α-helix content, representing the un-folding conformation, resulted in the decreased activity. The molecular docking further indicated that two sustainable hydrogen bonds were generated between astragalin and α-glucosidase residue Ser-88 and Tyr-133. The main driving forces to form the astragalin-α-glucosidase complex were the van der Waals force and hydrogen bond. Astragalin at a concentration of 80 µg/mL obtained the best hypoglycemic effect by activating the Adenosine 5′-monophosphate (AMP)-activated protein kinase (AMPK) signaling pathway. Conclusions: This study provides new insights into the potential utilization of astragalin-rich foods in the improvement of diabetes mellitus.

## 1. Introduction

Human physical and mental health has been seriously affected by metabolic diseases caused by modern lifestyle changes. At present, diabetes, together with cardiovascular diseases and cancer, has become one of the major chronic non-hereditary diseases threatening human health [1,2]. Glucose metabolism disorder is the main pathological feature for diabetes, and continuous hyperglycemia will cause cardiovascular and neurological complications. The mulberry leaf is a classic food material used in traditional Chinese medicine to treat diabetes, which has a long-term practical basis in folk health care and clinical practice [3]. Modern scientific experiments also confirmed that flavonoids, polysaccharides, and polyhydroxyalkaloids in mulberry leaves were important material bases for its hypoglycemic effect [4,5].

Astragalin (kaempferol-3-*O*-β-d-glucopyranoside) is an active natural flavonoid ingredient extracted from various traditional medicine plants, such as *Morus alba* L. [4], *Moringa oleifera* L. [6], *Astragalus hamosus* [7], and *Eucommia ulmoides* L. [8]. It obtains various biological activities such as antioxidant [9], anti-inflammatory [10], and hypnotic effects [11]. Astragalin improves airway thickening and alveolar destruction by blocking airway allergic inflammation, thereby antagonizing asthma [11]. As one of the main flavonoids from *Morus alba* (both leaves and fruit), astragalin increased endogenous estrogen and progesterone in aged female rats [12]. However, whether astragalin plays a role in the hypoglycemic effect of mulberry leaves needs further exploration.

The inhibitors inhibited the activity of α-glucosidase in the small intestine and slowed down the breakdown of polysaccharides into monosaccharides and the subsequent absorption, resulting in reduced insulin secretion in pancreatic tissue and alleviated insulin resistance [13]. Currently, the α-glucosidase inhibitors commonly applied in the adjuvant treatment for type-2 diabetes include miglitol, voglibose, and acarbose [1]. However, serious side effects were accompanied by the long-term use of these drugs [14]. Fortunately, dietary supplementation of natural active products with α-glucosidase inhibitory activity, reducing or eliminating adverse secondary side effects, has been proven to be an effective method in controlling hyperglycemia.

Adenosine 5′-monophosphate (AMP)-activated protein kinase (AMPK, EC 2.7.11.31), a heterorimetic protein composed of three subunits: alpha (63 kD), beta (30 kD), and gamma (37–63 kD) and belonging to serine/threonine protein kinase, plays a positive role in multiple signaling pathways regulating glucose metabolism homeostasis [15]. AMPK is mainly regulated by changes in the adenosine monophosphate/triphosphate (AMP/ATP) ratio and creatine/phosphocreatine ratio in cells [16]. Under the condition of hypoxia, glucose deficiency, and muscle activity, cells suffer from stress response characterized by ATP depletion, leading to the increase in the AMP/ATP ratio and the activation of AMPK [17]. AMPK activation stimulates the glucose uptake in muscle, fat, liver, pancreas and other organs, and inhibits endogenous glucose production, which plays an active role in preventing diabetes and improving metabolic syndrome [18].

The present study further compared the α-glucosidase inhibitory activity of the main phenolic compounds in mulberry leaves. In addition, the most active compound, astragalin, was selected for the inhibitory mechanism analysis. Furthermore, a high-glucose HepG2 cell model was established to reveal the pathway through which astragalin regulated glucose homeostasis. To the best of our knowledge, no study has reported the inhibition mechanism of astragalin against α-glucosidase. The results of this research will enrich the theoretical system of the substance basis for mulberry leaves’ hypoglycemic effect.

## 2. Materials and Methods

### 2.1. Materials

Acarbose, α-glucosidase, and p-nitrophenyl-α-d-glucopyranose (p-NPG) were purchased from Yuanye Biotechnology Co., Ltd., Shanghai, China. Standards such as astragalin, rutin, hyperoside, quercetin, gallic acid, gentic acid, chlorogenic acid, vanillic acid, caffeic acid, syringic acid, epicatechin, catechin, and benzoic acid were purchased from Qiyun Biotechnology Co., Ltd., Guangzhou, China, with a purity of >98%. HepG2 cells were purchased from Yuchi Biotechnology Co., Ltd., Shanghai, China. High-glucose Dulbecco’s modified Eagle medium (DMEM), fetal bovine serum (FBS), nonessential amino acid (NEAA), trypsin, penicillin streptomycin dual antibody solution, phosphate buffer solution (PBS), and HBSS buffer solution were purchased from MIKX Biotechnology Co., Ltd., Shenzheng, China. Glucose, Total cholesterol (TC), and total triglyceride (TG) kits were purchased from Jiancheng Bioengineering Research Institute, Nanjing, China. The other reagents were all analytical grade with a purity of >99.8%.

### 2.2. α-Glucosidase Inhibitory Activity

The inhibition activity was carried out with the help of the substrate p-NPG, based on a previous study with slight modifications [19]. α-glucosidase solution (1 U/mL) was prepared with 0.1 mol/L PBS 50 µL, together with atragalin (112, 224, 336, 448, and 560 mM) or acarbose (0.64. 1.28, 1.92, 2.56, and 3.2 mM) in a series of dilution concentrations, 50 µL, were added to a 96-well plate and incubated at 37 °C for 10 min. Then, 50 µL of 2 mmol/L p-NPG was added continually and incubated at 37 °C for another 20 min. Further, the enzyme activity was reduced by placing the well plate in an ice water bath for 5 min. Finally, 50 µL of 1 mol/L Na_2_CO_3_ was added to terminate the reaction, and the absorbance values were measured at 405 nm. The inhibition rates of α-glucosidase were calculated according to Formula (1), and the half maximal inhibitory concentrations (IC_50_) were obtained by nonlinear regression fitting.

Enzyme activity inhibition rate (%) = [1 − (Aa − Ab)/(Ac − Ad)] 100%(1)

Aa: sample group absorbance; Ab: sample background absorbance (equal volume buffer instead of enzyme solution); Ac: control group absorbance (equal volume buffer instead of sample solution); and Ad: control background absorbance (equal volume buffer instead of sample and enzyme solution).

### 2.3. Multi-Fluorescence Spectroscopy

Astragalin in different concentrations (0, 10, 20, 50, 80, 100, 120, and 150 mg/L) were mixed with α-glucosidase (1 U/mL), and incubated for 5 min at three temperatures (298 K, 304 K, and 310 K). The mixture was scanned using fluorescence spectroscopy (SpectraMax i3x, Molecular Devices, LLC., San Jose, CA, USA). The excitation wavelength was set at 280 nm, and the emission wavelength at 300–500 nm, with excitation and emission slit widths of 5 nm. Then, the following Stern–Volmer dynamic collision quenching equations were applied to calculate the fluorescence quenching parameters, including the quenching constants (*K_SV_*), quenching rate constant (*K_q_*), binding constant (*K_a_*), and binding site number (*n*) [20]. The equations are described below [1]:

(2) $$\frac{F_{0}}{F}=1+K_{SV}\left[Q\right]=1+K_{q}\tau_{0}\left[Q\right]$$

(3) $$lg\frac{F_{0}-F}{F}=lgK_{a}+nlg\left[Q\right]$$

*F* and *F*_0_ were the peak fluorescence intensities for α-glucosidase with and without astragalin, respectively; [*Q*] was the astragalin concentration; and *T*_0_ was the average lifespan of the protein (10^−8^ s).

### 2.4. FT-IR Measurements

The secondary conformation of α-glucosidase (1 U/mL) with or without sample treatment were detected with a Fourier transform infrared spectrometer (FT-IR) (VERTEX 7.0, BRUKER Co., Ltd., Bremen, Germany), by fitting the infrared amide type I curve (1600–1700 cm^−1^) [21]. The final concentration of astragalin was 100 µg/mL.

### 2.5. Molecular Docking

#### 2.5.1. Visual Docking

The molecular docking was performed with AutoDock Vina 1.1.2 software (Scripps Research Institute, Olson Lab, San Diego, CA, USA) [22]. The crystal structure of the α-glucosidase protein was retrieved from the PDB database (PDB id: 5NN4). The 3D structure of astragalin was downloaded from the pubchem database, and MMFF94 force filed was applied for energy minimization.

The α-glucosidase protein (EC 3.2.1.20) was processed with PyMol 2.5.2 prior to docking, and the docking box was set as Pymol plugin center_of mass.py. The ADFRsuite 1.0 (Scripps Research Institute, Olson Lab, San Diego, CA, USA) was used to convert into the PDBQT format required for AutoDock Vina 1.1.2 docking. The conformation with the highest output score was considered as the binding conformation, and PyMol 2.5.2 was used for the consequent visual analysis.

#### 2.5.2. Molecule Dynamics Simulation

Based on the conformation molecular docking as the initial structure, the full atom molecular dynamics simulation was carried out using AMBER 18 software (Scripps Research Institute, Olson Lab, San Diego, CA, USA) [23]. The antechamber module and the Hartree-Fock (HF) SCF/6–31G* of Gaussian 09 software were combined to calculate the charge of small molecules (Scripps Research Institute, Olson Lab, San Diego, CA, USA). In addition, the 2500-step steepest descent method and 2500-step conjugate gradient method were applied to optimize the energy of the system. The particle mesh Ewald (PME) method was employed to calculate electrostatic interactions, the SHAKE method was used to limit hydrogen atom bond lengths, and the Langevin algorithm was applied for temperature control. The collision frequency γ, the system pressure, and the integration step size was set to 2 ps^−1^, 1 atm, and 2 fs, respectively, and the trajectory was saved every 10 ps for subsequent analysis.

#### 2.5.3. MMGBSA Binding Free Energy

The molecular mechanics generalized born surface area (MM/GBSA) method was applied to calculate the binding free energy. A 90–100 nm MD trajectory was used for the calculation with the formula shown below [24]:

$${\Delta G}_{\mathrm{bind}}={\Delta G}_{\mathrm{complex}}-\left({\Delta G}_{\mathrm{receptor}}+{\;\Delta G}_{\mathrm{ligand}}\right)$$

(4) $$\;={\Delta E}_{\mathrm{internal}}+{\Delta E}_{\mathrm{VDW}}+{\Delta E}_{\mathrm{elec}}+{\Delta G}_{\mathrm{GB}}+{\Delta G}_{\mathrm{SA}}$$

ΔE_internal_: internal energy, including bond energy (E_bond_), angular energy (E_angle_), and torsional energy (E_torsion_), ΔE_VDW_: van der Waals interaction, and ΔE_elec_: electrostatic interaction. ΔG_GB_: polar salvation free energy; ΔG_SA_: non-polar salvation free energy.

### 2.6. Cell Experiment

#### 2.6.1. HepG2 Cell Culture

HepG2 cells at 10 to 40 generations were configured at a concentration of 4 × 10^5^/mL. The cells suspension 100 µL was inoculated into a culture plate with DMEM (10 mL) containing glucose 4.5 g/L, 10% FBS, penicillin (1000 U/mL), and streptomycin (10 mg/mL), and cultivated in a 37 °C, 5% CO_2_ incubator.

#### 2.6.2. HepG2 Cell Survival Rate

Astragalin (30, 50, 80, 100, and 200 µg/mL) was configured at different concentrations. Each sample was added to a 96-well plate with HepG2 cells and incubated in a 37 °C, 5% CO_2_ incubator for 24 h. After washing twice with PBS, the CCK-8 solution 100 µL was added, and incubated for another 4 h. The absorbance at 450 nm was measured using a Microplate reader (Bio TekGen5, BioTech Instruments, Co., Ltd., Winooski, VT, USA). The cells’ survival rates were calculated based on the formula as shown below:

Cell survival rate (%) = A samples/A black control × 100%(5)

#### 2.6.3. The Establishment of HepG2 High Glucose Model

When the cells were increased to about 80%, insulin at a concentration of 10^−5^ mmol/L was treated for 24 h to induce the formation of insulin-resistant cells [25]. Six experimental groups were set for the comparison, namely, black (normal control without insulin treatment), model (insulin treatment control), acarbose (5 µg/mL positive control with insulin treatment), and astragalin sample groups (20, 80, and 200 µg/mL, with insulin treatment). After the treatment of 24 h with different samples, the glucose, TC, and TG content in the HepG2 cell supernatant was measured with glucose, TC, and TG assay kits.

#### 2.6.4. Western Blotting

The proteins in the above six cell sample groups (black, model, acarbose, and astagalin 30, 80, and 200 µg/mL) were extracted with a total protein extraction buffer, and the concentration was detected with bicinchoninic acid (BCA) assay kits. Protein samples of 20 µg were separated by sodium dodecyl sulfate polyacrylamide gel electrophoresis in the Bio-Rad electrophoresis apparatus (Liuyi Biotechnology Co., Ltd., Beijing, China), and then transferred to polyvinylidene fluoride membranes. The electrophoretic proteins were sealed with 5% skimmed milk at room temperature for 1 h, and the membranes were incubated overnight with primary antibodies, including AMPK, phospho-AMPK (p-AMPK), acetyl CoA carboxylase (ACC, EC 6.4.1.2), and glucose transporter 4 (GLUT4) at 4 °C. After washing the membrane with phosphate-buffered saline tween-20, the membranes were incubated with secondary antibodies at room temperature for 1 h.

### 2.7. Statistical Analysis

The experiments were carried out in triplicate. The data analysis and figure drawing were processed with a combination of SPSS 22, Microsoft Excel 2010, Origin 8.0, and GraphPad Prism 9 software. The differences were considered significant when *p* < 0.05, and examined though a one-way analysis of variance (ANOVA).

## 3. Results and Discussion

### 3.1. The α-Glucosidase Inhibitory Effect of the Main Polyphenols in Mulberry Leaves

**Figure 1:**
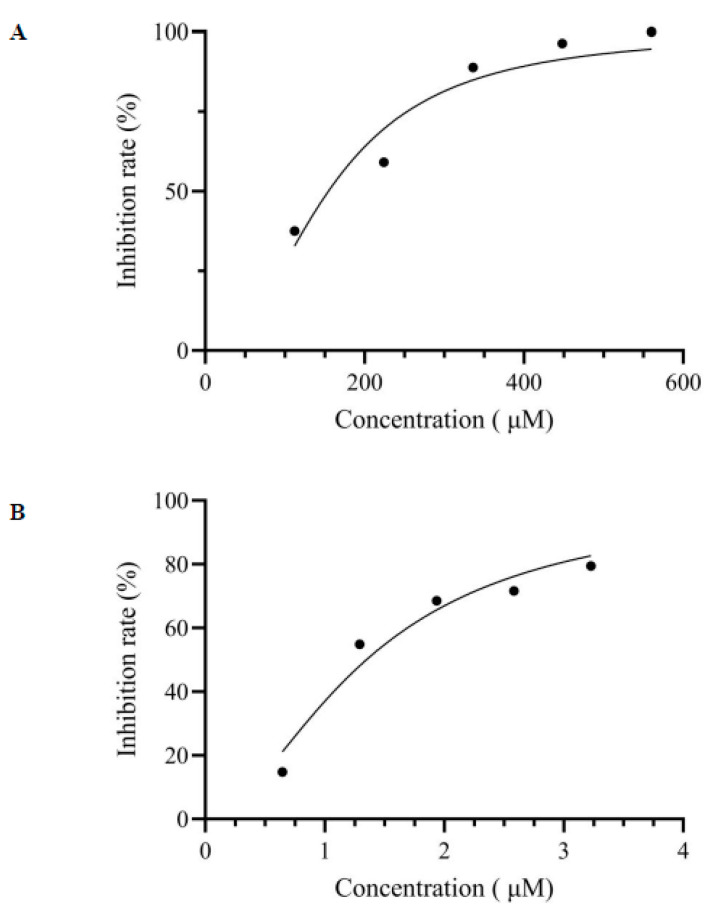
The inhibition rate for α-glucosidase under different concentrations of inhibitor. (**A**) Astragalin; (**B**) acarbose.

The main polyphenols in mulberry leaves included astragalin, rutin, chlorogenic acid, benzoic acid, epicatechin, catechin, vanillic acid, etc., which had been reported by our previous research [4]. The results of the present study comparing their α-glucosidase inhibition indicated that the activity of astragalin was the highest, with an IC_50_ value of 154.5 µM (Figure 1A), while the IC_50_ values of the other main polyphenols from mulberry leaves were all greater than 500 µM. Although the activity of astragalin was weak compared to acarbose (1.35 µM) (Figure 1B), it obtained a wide range of plant sources and safety. The activity of astragalin was superior to that of the reported compounds rutin, isoquercetin [26], 2,4-dimethoxy-6,7-dihydroxyphenanthrene [27], phlorizin [1], and batatasin I [28], with IC_50_ of 198 µM, 185 µM, 400 µM, 0.98 mM, and 2.55 mM, respectively. In addition to astragalin, the alkaloids such as 1-Deoxynojirimycin and its derivatives in mulberry leaves exhibited a strong α-glucosidase inhibitory activity, thereby exerting a hypoglycemic activity [5]. The hypoglycemic effects of astragalin were verified in streptozocin-induced diabetic mice, which improved the glucose tolerance and reduced the level of glucose [29]. In addition, astragalin decreased the over-expression of the vascular endothelial growth factor in Müller cells, and alleviated diabetic retinopathy caused by high glucose [30]. Meanwhile, the α-glucosidase inhibition and hypoglycemic activity mechanism were indeed lacking.

### 3.2. Binding Mechanism and Properties by Fluorescence Spectroscopy

**Figure 2:**
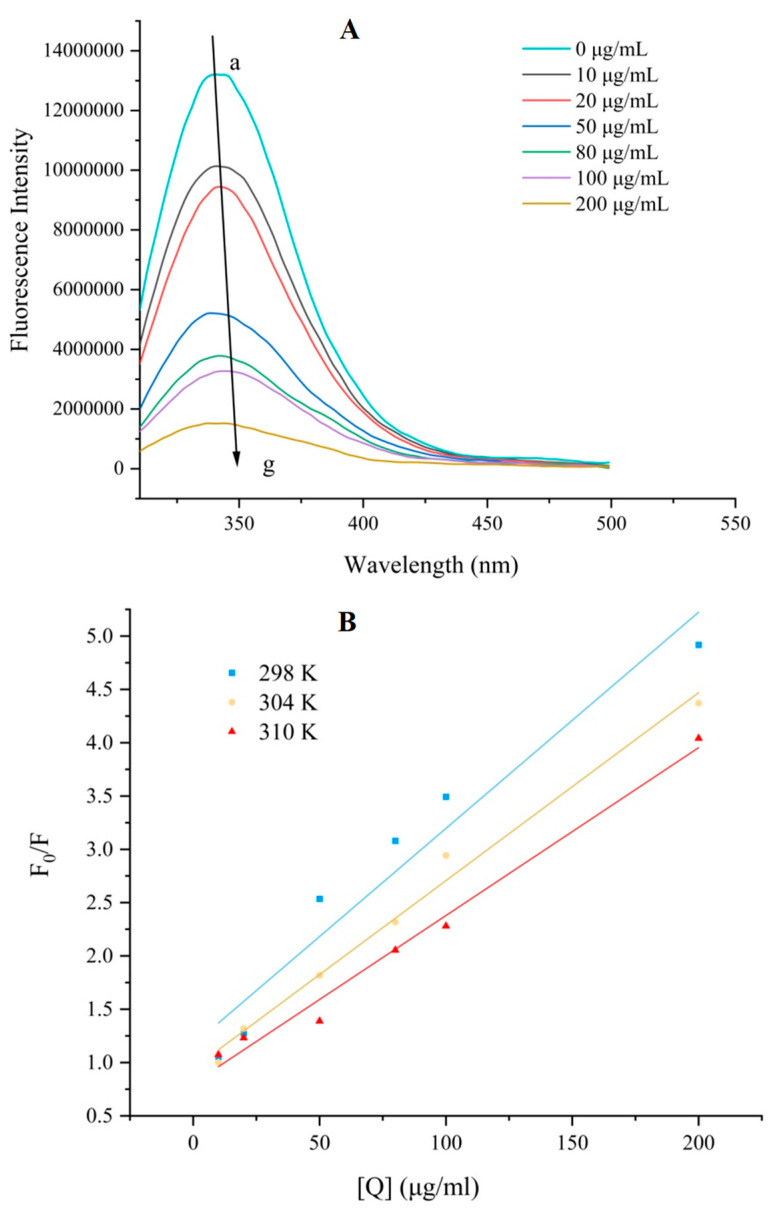
Fluorescence spectra of α-glucosidase with astragalin. (**A**) Fluorescence curves at 298 K in the present of increasing concentrations, (**B**) Stern–Volmer plots at different temperatures. Note: a–g represented the concentration of 0-200 µg/mL.

Dynamic quenching is formed by the collision between quencher and molecules-excited fluorescence, while static quenching is the formation of a complex through binding [31]. Proteins containing aromatic amino acids, such as tryptophan (try), tyrosine (tyr), and phenylalanine (phe), emit fluorescence at an excitation wavelength of 280 nm, while the intrinsic fluorescence intensity decreased when interacting with quenchers [32]. The free α-glucosidase had a maximum fluorescence emission peak around 346 nm, the fluorescence intensity decreased dose-dependently with the increase in astragalin concentration, and the maximum emission wavelength increased to 349 nm (Figure 2A). This red-shift phenomenon was caused by the unfolding of the α-glucosidase structure during the interaction with astragalin, exposing the florescent residues (try, tyr, and phe) to a more hydrophilic environment [33]. The results are consistent with previous studies [1,19], and astragalin induced microenvironment changes in the fluorophore of α-glucosidase.

The Stern–Volmer curves of astragalin with α-glucosidase showed a good linear relationship (Figure 2B), and the *K_SV_* value decreased with the increase in temperature (Table 1), indicating that the interaction between astragalin and α-glucosidase was supposed to be static quenching. The maximum diffusion collision quenching constant of the quencher for biomolecules is 2.0 × 10^10^ Lmol^−1^ s^−1^. Under three temperature conditions, the *K_q_* values of astragalin with α-glucosidase were much higher than 2.0 × 10^10^ Lmol^−1^ s^−1^, proving the static quenching interaction mechanism and the formation of a complex with α-glucosidase [1,34]. The number of binding sites (*n*) for astragalin with α-glucosidase were close to one under three different temperatures, indicating a 1:1 molar ratio of interaction and possessing one binding site. The *K_a_* values of astragalin with α-glucosidase decreased with the increase in temperature (298–310 K), indicating a reduction in the complex stability [22].

### 3.3. FT-IR Analysis

**Figure 3:**
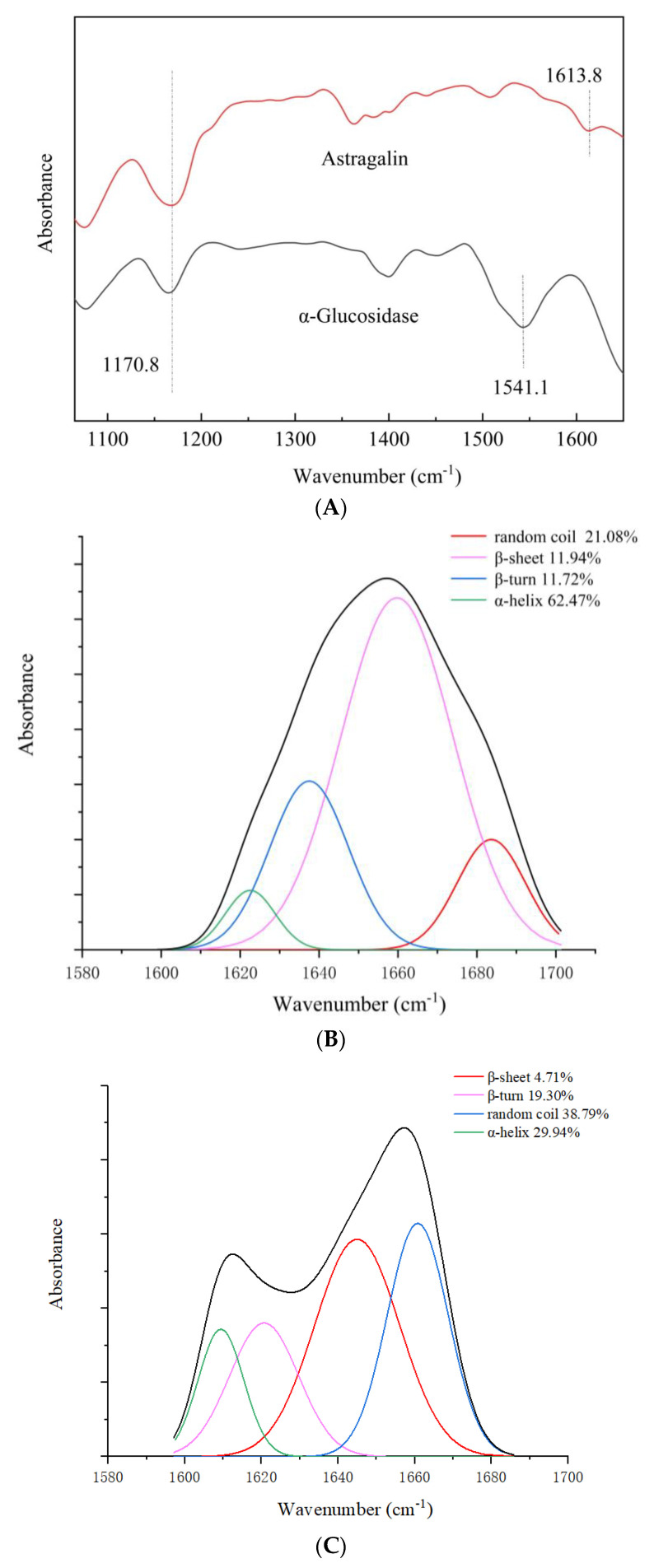
The FT-IR spectra of α-glucosidase with astragalin. (**A**) FT-IR curves, (**C**) α-glucosidase in the absence of astragalin (1 U/mL), and (**B**) α-glucosidase with astragalin (100 µg/mL). Note: the black line represented the overall secondary structure total fluorescence structure with a value of 100%.

The protein secondary structure could be altered by binding with phenolic compounds [13]. The effect of astragalin on the α-glucosidase structure was further investigated using FT-IR spectroscopy, which indicated that the binding of astragalin with α-glucosidase changed the secondary structure of the protein, thereby affecting the enzyme activity. The amide I band (1600 cm^−1^–1700 cm^−1^, derived from the stretching vibration of –C=O) is more sensitive to the protein secondary structural changes than the amide II band (1500 cm^−1^–1600 cm^−1^, mainly consisting of C-N stretching vibration and N-H bending vibration peaks) [35]. As shown in Figure 3A, the peak intensity was significantly enhanced in the astragalin/α-glucosidase complex; the absorption peak at 1541.1 cm^−1^ in the amide II band in α-glucosidase was enhanced significantly with the treatment with astragalin.

The relative content of the amide I band in the protein secondary structure was determined using a curve-fitting protocol coupled with second-derivative resolution enhancement (Figure 3B,C). The results indicated that, with the addition of astragalin, the α-helix, β-turn, β-sheet, and random coil of α-glucosidase changed from 62.47%, 11.72%, 11.94%, and 21.08%, to 29.94%, 19.30%, 4.71%, and 38.79%. The α-helix content of the α-glucosidase secondary structure was significantly decreased from 62.47% (Figure 3B) to 29.94% (Figure 3C). The α-helix around the catalytic site of the enzyme was confirmed to play a crucial role in stabilizing the activity. It was speculated that the decrease in the α-helix content was related to the decrease in the α-glucosidase activity [36]. The results confirmed that astragalin induced the partial unfolding of the α-glucosidase conformation. The interaction between astragalin and the α-glucosidase −C=O group subunits resulted in the rearrangement of the α-glucosidase secondary structure, thereby reducing the α-glucosidase activity [33,37].

### 3.4. Molecular Docking Results for Astragalin with α-Glucosidase 

**Figure 4:**
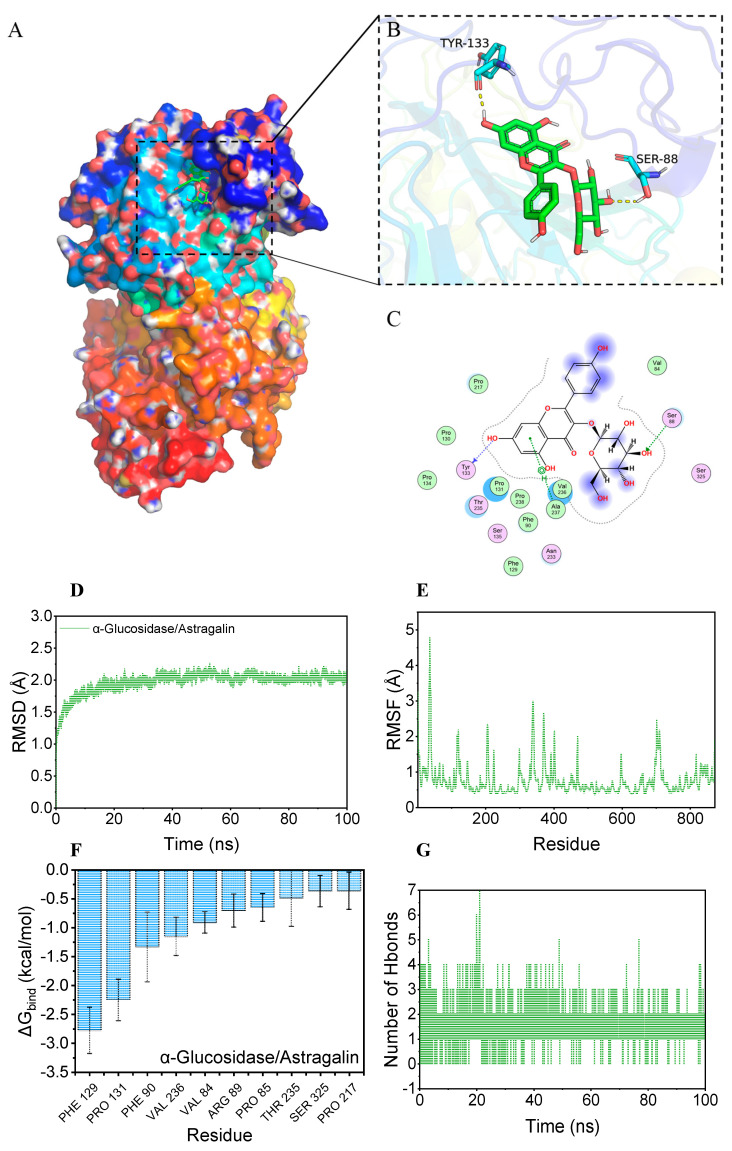
Binding diagram and dynamics simulation of α-glucosidase with astragalin. (**A**) Overall view of 3D binding diagram, (**B**) partial view; astragalin is represented by the green stick, amino acid residues of the binding site are represented by light blue, and hydrogen bonding is represented by the yellow dashed line. (**C**) 2D interaction diagram. (**D**) Root mean square deviation (RMSD), and (**E**) root mean square fluctuation value (RMSF) based on molecular dynamics simulation. (**F**) Top 10 amino acid residues contributing to the binding of α-glucosidase with astragalin. (**G**) The number of hydrogen bonds between astragalin and α-glucosidase changes during the molecular dynamics simulation.

Molecular docking was employed to further explore the binding mechanism of active molecules to macromolecular proteins [38]. The negative binding affinity indicated the possibility of binding; usually, a value less than −6 kcal/mol was considered to have a high binding probability. The binding affinity score for the astragalin/α-glucosidase complex was −7.238 kcal/mol based on molecular docking, indicating that astragalin had a good binding potential with α-glucosidase. The α-glucosidase active pocket was a hydrophobic cavity based on a previous report [39]. The visual binding complex formed by astragalin with α-glucosidase is exhibited in Figure 4. Astragalin was bound to the internal active pocket of the α-glucosidase protein (Figure 4A–C). The active pocket was constructed by the surrounding of Val-84, Pro-217, Pro-130, Pro-131, Val-236, Ala-237, Pro-238, Ser-88, Tyr-133, and Ser-325 amino acid. Among them, astragalin underwent hydrophobic interactions with Val-84, Pro-217, Pro-130, Pro-131, Val-236, Ala-237, and Pro-238 on the active binding site, while forming hydrogen bonding interactions with Ser-88 and Tyr-133. These interactions were supposed to be the main reason for the stable binding of the astragalin with α-glucosidase [40]. This visual result further confirmed one binding site in the fluorescence spectroscopy.

During the molecular dynamics simulation, a high root mean square deviation (RMSD) represented intense fluctuation and motion for the docking complex [41]. As shown in Figure 4D, there was no significant RMSD change for the astragalin/α-glucosidase complex, indicating the high stability of the complex and the correction of the simulation parameters. The root mean square fluctuation (RMSF) reflected the flexibility of the protein [36]. The flexibility decreased when the protein combined with active molecules, thereby achieving complex stabilization and activity inhibition. As shown in Figure 4F, except for a small part of the α-glucosidase protein, the RMSF of the complex was sustained within 2 angstroms, indicating that the main protein complex structure obtained a very high rigidity, which might be achieved by the combination with astragalin.

Based on the trajectory of the dynamics simulation, the binding energy was calculated using the MM-GBSA method, which more accurately reflected the binding effect between molecules and target proteins [22]. The binding energy (ΔG_bind_) of astragalin/α-glucosidase was −21.95 ± 1.21 kcal/mol (Table 2), indicating a strong binding affinity. The main contributions to the binding were the van der Waals energy and electrostatic energy. The top 10 amino acids that contribute to the binding, namely Phe-129, Pro-131, Phe-90, Val-236, Val-84, Arg-89, Pro-85, Thr-235, Ser-325, and Pro-217, respectively, which were the key amino acids, and the corresponding residue energy are exhibited in Figure 4F. The hydrogen bonds number between astragalin and α-glucosidase was monitored during a 100 ns dynamics simulation, which was the strongest noncovalent interaction. The number of hydrogen bonds ranged from 0 to 7, most of the time being concentrated at 2, indicating that hydrogen bonding contributed to the stable binding of astragalin and α-glucosidase [38].

### 3.5. HepG2 Cell Experiments

**Figure 5:**
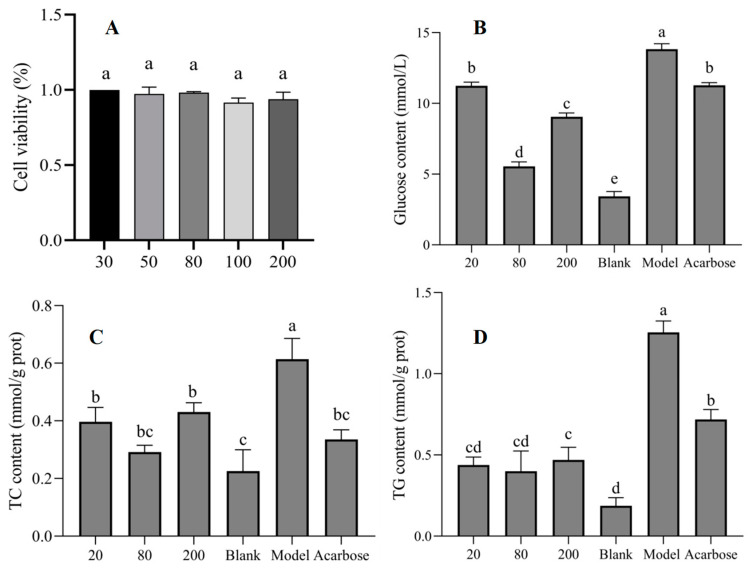
Effect of astragalin on glucose homeostasis regulation. (**A**) CCK-8 under different treatment concentrations (µg/mL), (**B**) intracellular glucose level, (**C**) intracellular TC level, and (**D**) intracellular TG level. Note: groups with different characters (a, b, c, d, and e) represented significant differences.

The liver is one of the important sites for glucose metabolism; the regulatory mechanisms of glucose metabolism were extensively studied by many researchers, based on liver cell models [25,42]. The CCK-8 results for astragalin indicated that concentrations from 0–200 µg/mL were within the safe range on HepG2 cells (Figure 5A). The high glucose model was induced successfully by insulin in HepG2 cells, where the glucose level was increased significantly from 3.43 mmol/L (Black) to 11.28 mmol/L (Model). In the model group, insulin resistance was induced, leading to the decrease in the glucose consumption and the large accumulation of glucose in the cells [25]. At concentrations of 20, 80, and 200 µg/mL, astragalin reduced the glucose level in the cells, and the activity was the strongest at a concentration of 80 µg/mL, which was superior to that of acarbose as positive control drug (5 µg/mL) (Figure 5B). In addition, the content of TC and TG also significantly increased in the model group, and astragalin under different concentrations reduced the levels of TC and TG in high-glucose HepG2 cells (Figure 5C,D).

**Figure 6:**
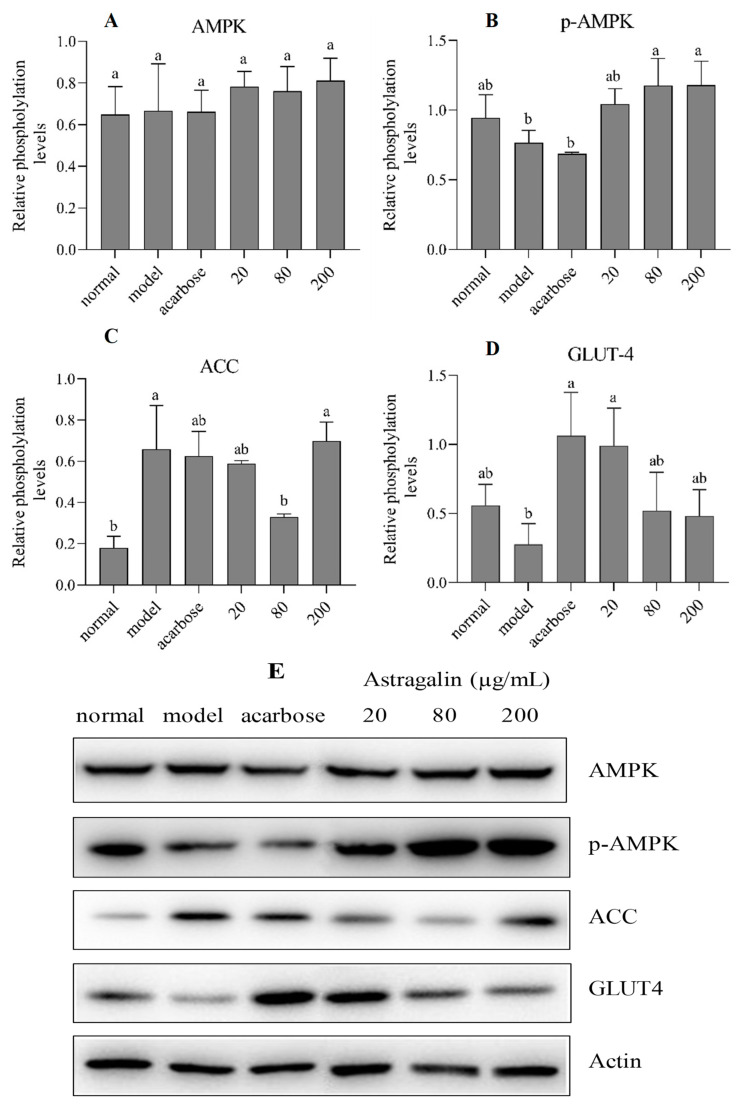
Expression level of the proteins related to glucose (**A**) AMPK, (**B**) p-AMPK, (**C**) ACC, (**D**) GLUT4, and (**E**) Grayscale image for the Western blot. Note: groups with different characters (a or b) represented significant differences.

The results from the Western blotting (Figure 6) show that astragalin under concentrations of 80 and 200 µg/mL up-regulated the protein expression level of p-AMPK protein in high-glucose HepG2 cells with the activation of the AMPK pathway (Figure 6A,B). The activity of the rate-limiting enzyme in liver glucose metabolism was closely related to the activation of the AMPK pathway, which stimulated the phosphorylation of phosphofructose-2-kinase to promote glycolysis, and reduced the expression of fructose-1,6-diphosphatase to inhibit gluconeogenesis [43]. In addition, the acetyl CoA carboxylase (ACC) protein expression level was significantly down-regulated in the 80 µg/mL concentration group (Figure 6C), which might contribute to the highest hypoglycemia activity in this group. The increase in ACC expression promoted fat synthesis by producing malonyl-CoA, which is utilized by fatty acid synthase to produce palmitic acid [3]. In addition, the protein expression level of glucose transporter 4 (GLUT4) significantly increased in the 20 µg/mL group (Figure 6D), indicating a significant increase in glucose transport levels, thereby enhancing glucose consumption and glycogen synthesis [25,44]. The action pathway for astragalin was similar to that of the previously reported phenolic compounds. Resveratrol activated the downstream ACC though AMPK, thereby improving the glucose level and inhibiting liver fat accumulation [45]. *p*-coumaric acid promoted the p-AMPK protein expression level in L6 skeletal muscle cells and increased ACC phosphorylation, thereby reducing glucose levels and promoting the β-oxidation of fatty acids [46]. In addition to polyphenols, recent studies have found that many compounds could activate the AMPK signaling pathway. Glutamine prevented intervertebral disc degeneration by decreasing AMPK lactylation and inhibiting glycolysis [47]. Fingolimod inhibited apoptosis in type 2 diabetic mice via activating the AMPK/mammalian target of the rapamycin (mTOR) signaling pathway [48].

## 4. Conclusions

Overall, astragalin obtained a potential hyperglycemic activity as evidenced by the α-glucosidase inhibition activity and the reduction in the glucose level in HepG2 cells. The results indicated that astragalin was an effective α-glucosidase inhibitor with the IC_50_ of 154.5 µM, which was the lowest concentration among the main polyphenols from mulberry leaves. The intrinsic fluorescence of α-glucosidase was quenched by the interactions with astragalin in a single inhibition site through a static quenching mechanism. Meanwhile, the interactions changed the micro-environments and conformation of α-glucosidase, and the decrease in α-helix content, representing the secondary structure un-folding conformation, resulted in the decreased activity. The exact binding site of astragalin on α-glucosidase was displayed by molecular docking. Astragalin at a concentration of 80 µg/mL obtained the best hypoglycemic effect on the high-glucose HepG2 cell model, which exerted effects by activating the AMPK signaling pathway. Our research further enriched the theoretical basis for the design of hypoglycemic functional foods with mulberry leaves. Currently, fresh vegetables, tea, and hypoglycemic functional health foods made from mulberry leaves have been widely recognized by consumers. However, further research and development are still needed for hypoglycemic products containing astragalin. In our opinion, research on the digestion, absorption, intestinal metabolism, and functional targets of astragalin will help promote the industrial application of plant/pure astragalin.

## Figures and Tables

**Table 1:** The quenching constants (*K_SV_*), binding constants (*K_a_*), and binding site (*n*) for the interaction of astragalin with α-glucosidase.

T (K)	*K_SV_* (×10^3^ Lmol^−1^)	R ^a^	*K_q_* (×10^11^ L mol^−1^)	*K_a_* (×10^5^ L mol^−1^)	*n*	R ^b^
298	9.093	0.97	9.093	4.29	1.238	0.98
304	7.903	0.99	7.903	3.01	1.1905	0.96
310	7.061	0.97	7.061	2.56	0.8446	0.96

Note: R ^a^ is the correlation coefficient for the *K_SV_* values; R ^b^ is the correlation coefficient for the *K_a_* values.

**Table 2:** Binding free energies and components predicted by MM/GBSA (kcal/mol).

System Name	Alpha-Glucosidase/Astragalin
ΔE_vdw_	−29.99 ± 2.64
ΔE_elec_	−23.18 ± 2.81
ΔG_GB_	35.65 ± 2.66
ΔG_SA_	−4.43 ± 0.44
ΔG_bind_	−21.95 ± 1.21

ΔE_vdW_: van der Waals energy; ΔE_elec_: electrostatic energy; ΔG_GB_: electrostatic contribution to salvation; ΔG_SA_: non-polar contribution to salvation; and ΔG_bind_: binding free energy.

## Data Availability

The original contributions presented in this study are included in the article. Further inquiries can be directed to the corresponding author.
